# Realistic Facial Expression of Virtual Human Based on Color, Sweat, and Tears Effects

**DOI:** 10.1155/2014/367013

**Published:** 2014-07-17

**Authors:** Mohammed Hazim Alkawaz, Ahmad Hoirul Basori, Dzulkifli Mohamad, Farhan Mohamed

**Affiliations:** ^1^Faculty of Computing, Universiti Teknologi Malaysia, 3rd Floor, N28A, 81310 Johor Bahru, Johor, Malaysia; ^2^Interactive Media and Human Interface Lab., Department of Informatics, Faculty of Information Technology, Institut Teknologi Sepuluh Nopember Surabaya, Surabaya 60111, Indonesia; ^3^Faculty of Computing and Information Technology Rabigh, King Abdulaziz University, Rabigh, Makkah 21911, Saudi Arabia

## Abstract

Generating extreme appearances such as scared awaiting sweating while happy fit for tears (cry) and blushing (anger and happiness) is the key issue in achieving the high quality facial animation. The effects of sweat, tears, and colors are integrated into a single animation model to create realistic facial expressions of 3D avatar. The physical properties of muscles, emotions, or the fluid properties with sweating and tears initiators are incorporated. The action units (AUs) of facial action coding system are merged with autonomous AUs to create expressions including sadness, anger with blushing, happiness with blushing, and fear. Fluid effects such as sweat and tears are simulated using the particle system and smoothed-particle hydrodynamics (SPH) methods which are combined with facial animation technique to produce complex facial expressions. The effects of oxygenation of the facial skin color appearance are measured using the pulse oximeter system and the 3D skin analyzer. The result shows that virtual human facial expression is enhanced by mimicking actual sweating and tears simulations for all extreme expressions. The proposed method has contribution towards the development of facial animation industry and game as well as computer graphics.

## 1. Introduction

Definitely, the face of human is the most significant art object and central aspect of interaction. The proportions and the expressions of the face are important to identify the origin, the emotional tendencies, and the health qualities and are often fundamental to the human social relations. Different types of essential information are noticeable on our faces. Communications of human reactions act as adaptive resolution [1–3]. Indeed, the human reactions play a substantial role in social contacts where the participants constantly perceive and react to each other. Besides, the facial expression via emotions, vocalization, and motions in gestures influences our interaction processes. Various autonomic mediated signals such as coloration change due to blushing, blanching, and bulging of arteries emerged with emotions. Additional detectable changes involving piloerection, offensive sweat, tears, and crying also majorly affect facial appearances [4].

Considerations of autonomically mediated signals in the process of building embodied virtual agents or humans which effectively and naturally communicate emotions with real humans are the key issue [5]. Virtual characters in games and simulations look so realistic provided they own detailed geometry and superior animations. Recently, the advancements in motion capture and automatic blending methods facilitated a strong display of facial and body motions. Truly, the applications of virtual characters in 3D environments reveal emotional connection with the user brought tremendous benefits. Therefore, a correlation between the emotions based virtual character and the user needs to be established for realistic computer animations.

Though, virtual characters display different body motions [6] and facial expressions [7] but their emotional ranges are relatively limited. For instance, extreme expressions such as laughing out loud, screaming in anger, or crying in pain are hardly visible in games. Conversely, in the movies such expressions are regularly used to attract the audience. In animation, creation of highly accurate human face demands dedicated effort, time, and skills. Alternatively, realistic animation is absolutely needed because the facial expression corresponds to human feelings and mental states. Generating credible facial animation is very vital towards the understanding of emotions [4, 8].

In physiological arousal, the emotions related to the relationship changes in the extreme light of physiological processes occur inside the human body. These changes are already included in the variations of sweat glands and salivary activity to generate realistic expressions. The levels of some neurotransmitters inside the brain, metabolism, muscle strain, digestive system, and consequently the revised digestion are responsible for such alterations. Other physiological processes which cause these changes are the facial color, the piloerection, the facial expressions, and additional signs of emotion [9]. Motions also represent behavioral responses and the emotion is the key to human expression in terms of feelings or participations. These expressions range from screaming to crying to verbal that characterizes laugh or smile. Besides the postures and other physical gestures the other common emotion sign is the voice tone [9]. All kinds of fear and happiness may not cause sweating or tears in humans, due to emotion intensity level. Despite many efforts, no relationship between realistic facial color generation and oxygenation/deoxygenation effects of hemoglobin is acknowledged. Therefore, it is important to develop a comprehensive animation model for 3D avatar by integrating the effects of sweat, tears, and color to create realistic facial expressions of sad, fear, happy, and angry.

We developed a new method for creating extreme expression in 3D facial animation containing colors, sweat, and tears. Physical properties of muscles with sweating and tears initiators are incorporated in the model. FACS with AUs is used to generate realistic facial expressions for sadness, anger with blushing, happiness with blushing, and fear. These extreme facial expressions are mimicked using particle system and SPH methods. The realism of virtual human is significantly enhanced. A correlation between realistic extreme facial expression for virtual human emotions and the effects of facial skin color, tears, and sweat is established.

## 2. Related Work

Generation of virtual human via computer animation model is in exponential progression [10]. Currently, the natural interaction capability of an avatar is guided by the developments of artificial intelligence, diverse sensing technology, and advanced computer graphics [11]. The realistic facial animation applications offered an opportunity to bring facts and expressions of human to the social reality. More significantly, the facial animation is advanced in the field of computer games, medicine, multimedia, and movies. It is realized that the virtual characters in computer games and simulations must relate to real situations with greater geometric details. The actions and approaches for automatic blending of the body together with facial motions are well recognized [12].

The task is becoming challenging because the animations at each event require the synchronization of facial components in speech which involve facial bones, muscles, and lips. Animation of highly accurate human face is complicated. Consequently, inclusion of several notable attributes in the facial expressions to precisely visualize the people feelings and mental states is essential. The facial action coding system (FACS) of is used to process and describe the facial behaviors via every facial muscle [13]. For each facial muscle, it is a standard practice to change the appearances based on the anatomy analysis of human facial muscles conduct together with the tongue and jaw motion [14]. Facial anatomy deals with the changes in facial expression caused by their actions. Unlike muscle-based techniques, FACS works on facial actions and facial behaviors by studying the muscle actions rather than the muscle itself. Understanding the origin of facial expressions is complicated because they originate from cooperative effects of many muscles. Moreover, the muscle configurations are highly multifarious [15, 16]. Facial AUs are introduced to support the actions of facial muscles. When an expression is generated, the involved muscles functions are intricate and vice versa. Each muscle is divided into two or more AUs to completely explain autonomous actions of muscle parts. FACS classifies the human face into forty-six AUs because each unit embodies individual muscle action or groups of muscles describe a single facial state. Principle of each AU cannot be dealt with in minor units. Exact taxonomy of AUs on the face is to mimic entire facial muscle actions and the blending of different AUs yields diverse facial expression. For example, merging the AU4 (brow raiser), AU15 (lip corner depressor), AU1 (inner brow raiser), and AU23 (lip tightener) produces sad appearance. FACS explains true facial expressions built by all possible facial animation units prepared by the human face gauging head and eye point only. The delicate side of FACS accounts for obvious facial changes. It totally neglects the trivial changes such as insignificant transformation under skin that affect facial expression so it become difficult to be distinguished [15, 17]. Effectively, FACS is exploited to differentiate the parts of facial actions and to generate the four basic expressions such as happy, fear, sad, and angry.

Generating 3D human faces is exceedingly complex in computer graphics design [18]. Current technologies of facial animation synthesis are unable to generate realistic facial expressions containing basic emotions [19]. de Melo and Gratch simulated facial emotional expressions for blushing, sweating, tears of fear, anger, and wrinkles to express the physiological emotions in human [20]. They still relied on unrealistic texture based method which is a static one. Facial colors of humans manifest the virtual effect, emotional estimate, facial image, remote health care, and individual identification. Indeed, the colors of human face are regarded as one of the most peculiar forms of expressions [21].

Blushing is a common topic of psychological study which is proven to be an important facial cue which serves vital functions in an interpersonal communication [13]. The explanations on people blushing remain an unsolved issue among psychologists. Most people consider blushing in public as an uncontrollable response. Individuals do feel embarrassment when blushing in public. Furthermore, blushing is a symptom that made it even worse for people who suffer from social phobia [22]. Former studies focusing mainly on the geometric features of these alterations considered the facial surface animation including skin stretching and wrinkled structures only. However, the changes in hemoglobin concentration are known to alter the skin color. It may originate from the reaction of histamine or other skin conditions such as blushing and rashes. Generally, blushing is comprised of joy, shame, and anger. Regardless of its ability to convey emotion, the dynamic changes that occur in skin pigmentation are mostly ignored in the existing skin appearance models [23, 24]. Therefore, simulation of blushing for the expressions of anger and happiness is our special interest.

Sweating is primarily attributed to thermoregulation and emotional stress [25]. The latter is considered as emotional sweating which is physiologically manifested in the palm of hand, feet, axillae, and head [26, 27]. They may occur due to the exposure to fearful circumstances including shyness and social phobia [28]. We do simulation for sweating in the forehead due to fear using particle system and SPH method. Crying is usually associated with the experience of intense emotions of personal joy, separation, loss, failure, anger, discipline, guilt, and so forth [29]. Physiologically, crying is evident from the shedding of tears and a distinct noise. Several explanations are acknowledged for crying such as crying is perceived as a drops of physical effect in term of fluid that experienced by human in their life [30]. Alternatively, crying is viewed as attachment centered which describes it as an appeal for the protective presence of a parent [31]. For infants, crying is a call that draws attention to the caretakers for providing protection in urgent need like danger, breast feeding, and so on. Conversely, in adulthood crying continues to be a reaction of a loss that conveys an attachment message which seeks to trigger a reaction from its caretakers akin to spouse or friends. Therefore, two factors which can strongly influence the simulation of tears are the communicative function and the association of tears with strong emotions.

## 3. Methodology

**Figure 1:**
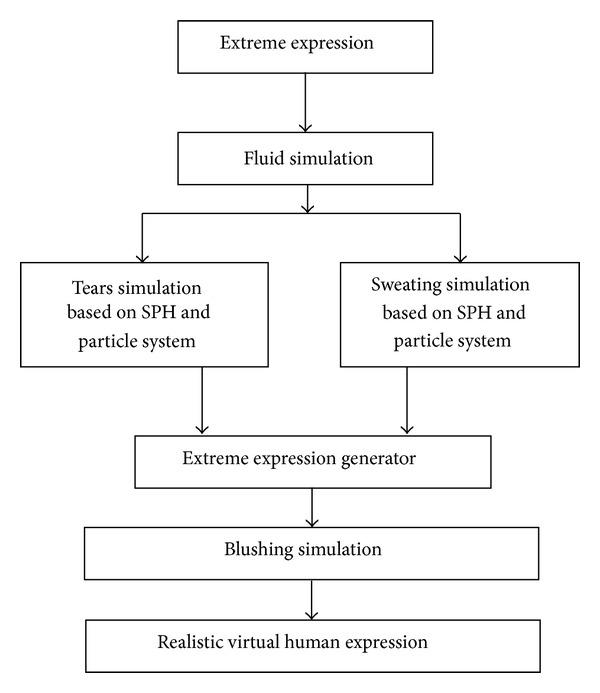
Research methodology.

Figure 1 displays the systematic design of the methodology. The approaches used to develop the computer graphics for facial animation to achieve the realism by generating actual sweating, tears, and blushing are highlighted. Various components of the method are briefly discussed hereunder.

### 3.1. Extreme Expression

Facial animation alone cannot simulate the extreme emotion of virtual human. Extreme expressions such as scared with sweating and happy until having tears are very strong emotional appearances. Therefore, supplementary elements including fluid mechanism (particle system) and SPH are required to provide these types of features. The facial animation is combined with these elements to perform the simulation.

### 3.2. Fluid Generator

This method creates a fluid simulation which is as realistic as touching tears. The art of creating small drops of rolling fluid requires a particle system which is certainly an efficient technique. The mixture of height area estimates per particle-based liquid generates another unit which is considered as a basic particle system reliable for producing splashes, spray, or water particles. Normally, this is not required to simulate particles system with one another but particles without intraparticle interaction. This simple particle can efficiently be simulated. It needs only a set of *N* particles 0 ≤ *i* ≤ *N* with masses *m*_*i*_, positions *x*_*i*_, velocities *v*_*i*_, and accumulated external forces *f*. These particles are usually prepared and placed in substantial positions and velocities before being simulated. Normally, an emitter cuts down the particles that have fixed particle rates on particular velocity in the same direction. It is also ensured that the particles are not only created but also disappear within a specific time interval. They simply fade out once maximum lifetime is attained. In addition, the uses of the lives to paint the particles are properly accounted for. Only per-particle forces exist without any trace of particle-particle interactions and the prevailing equation is decoupled into regular differential equations [32].

### 3.3. SPH Interaction for Particle System

The creation of the particles is more realistic for the simulation of tears or sweating if properties of SPH method including pressure and viscosity are properly implemented. The use of the SPH is much better than the Lennard-Jones interaction forces because of the presence of smoothness. The SPH method being used for stars simulation can be smoothen via smoothing kernels *W*(*r*). The kernel *W*(|*x* − *x*_*i*_|) is a scalar weight function near the *x*_*i*_ position of the particle I possessing exchange symmetry. The stability of kernel function obeys ∫*W*(|*x* − *x*_*i*_|)*dx* = 1. Following [33], we use the distinguished poly6 kernel given by

(1) $$\begin{array}{c} W\text{poly}6\left(r\right)=\frac{315}{64\pi d^{9}}\{\begin{array}{cc} {\left(d^{2}-d^{2}\right)}^{3} & 0\leq r\leq d\\ 0 & \text{otherwise}, \end{array} \end{array}$$

where *r* is the displacement that needs to be determined.

All elements are computed as an even density area in the individual positions using

(2) $$\begin{array}{c} P\left(x\right)=\sum m_{j}W\left(\left|x-x_{j}\right|\right), \end{array}$$

where *ρ*_*i*_ = *ρ*(*x*_*i*_) is the density of particle *i*. Kernels are normalized and the total mass of the system is computed as the integral of the density field using

(3) $$\begin{array}{c} \int p\left(x\right)dx=\underset{j}{\sum }\left(m_{j}\int w\left(\left|x-x_{j}\right|\right)dx\right)=\underset{j}{\sum }m_{j}. \end{array}$$

From the contaminants, the following gradient of the area is calculated by changing the kernel through their slopes:

(4) $$\begin{array}{c} A_{s}\left(x\right)=\underset{j}{\sum }m_{j}\frac{A_{j}}{P_{j}}W, \end{array}$$

(5) $$\begin{array}{c} \nabla A_{s}\left(x\right)=\underset{j}{\sum }m_{j}\frac{A_{j}}{P_{j}}\nabla W. \end{array}$$

In the Euclidian formulation, the fluids are described by a velocity *v*, density *ρ*, and a pressure *p*. The time evolution of these quantities is given by two equations in which the first one assures the conservation mass. Use of particles instead of a stationary power grid simplifies both equations significantly. The contaminants quantity is constant in terms of mass particle because of conservation. The first equation can completely be overlooked. The continuity equation is

(6) $$\begin{array}{c} \frac{\partial p}{\partial t}+\nabla \cdot \left(pv\right)=0. \end{array}$$

The Navier-Stokes equation which is based on momentum conservation is given by

(7) $$\begin{array}{c} p\left(\frac{\partial v}{\partial t}+v\cdot \nabla v\right)=-\nabla p+pg+\mu \nabla^{2}v, \end{array}$$

where *g* is an external body force and *μ* is the fluid viscosity. Secondly, the expression ∂*v*/∂*t* + *v* · ∇*v* on the left-hand side can be replaced by (*Dv*/*Dt*). The particles can move with the fluid due to the basic substantial derivative of the velocity which is just in the durational derivative of particles velocity. Therefore, the convective term *v* · ∇*v* becomes the trivial one for particle systems. The existence of three mass forces (N/m^3^) on the right-hand side related to the pressure (−∇*p*), gravitation (*ρg*), and viscosity (*μ*∇^2^ *v*) indicates that the change in momentum *ρ*(∂*v*/∂*t*) of the particles on the left-hand side is responsible for the acceleration:

(8) $$\begin{array}{c} a_{i}=\frac{\partial v_{i}}{\partial t}=\frac{f_{i}}{p_{i}}, \end{array}$$

where *v*_*i*_ is the constant velocity and *f*_*i*_ and *ρ*_*i*_ signify the body mass force and the density assessed at the location of particle *i*, respectively. We aim to evaluate the body forces via SPH method.

#### 3.3.1. Pressure

Application of the SPH method using (5) with the pressure term −∇*p* yields

(9) $$\begin{array}{c} f_{i}^{\text{pressure}}=-\nabla p\left(x_{i}\right)=-\underset{j}{\sum }m_{j}\frac{p_{j}}{p_{j}}\nabla w\left(\left|x_{i}-x_{j}\right|\right). \end{array}$$

Here the pressure is not symmetric per couple of contaminants interaction. The kernel gradient is zero at the center once the particle *i* utilized the pressure of particle *j* for computation and other evasions. Meanwhile, the unequal locations of these two contaminants make the pressure forces asymmetric. The solution of (9) is quite simple, stable, and fast to compute. We solve the following equation:

(10) $$\begin{array}{c} f_{i}^{\text{pressure}}=-\underset{j}{\sum }m_{j}\frac{p_{i}+p_{j}}{2p_{j}}\nabla w\left(\left|x_{i}-x_{j}\right|\right). \end{array}$$

Firstly, particles carrying 3 mass forces with respect to the position, velocity, and pressure are selected. Equation (2) gives the density at the position of the particle while the pressure is calculated from ideal gas law:

(11) $$\begin{array}{c} P=k\left(\rho -\rho_{0}\right), \end{array}$$

where *k* is the gas constant explicitly dependent on the temperature and *ρ*_0_ is the surroundings' pressure.

The pressure depends on the gradient from the force area, while the offset does not affect the pressure forces. However, the offset influences the gradient of the area smoothed by SPH and makes the mathematical simulation more stable. Nevertheless, the incompressibility is not strictly enforced as in the Eulerian situation. The minute variations in the density which produced the pressure forces are simply created afterward. This clearly controls the bouncy behavior in the fluid. The densities are obtained from the pressure forces by estimating the velocity area divergence via the SPH which is then used to fix the Poisson equation close to the particles [34].

#### 3.3.2. Viscosity

Using SPH, the asymmetric viscous force *μ*∇^2^ *v* is calculated from

(12) $$\begin{array}{c} f_{i}^{\text{viscosity}}=\mu \nabla^{2}v\left(x_{i}\right)=\mu \underset{j}{\sum }m_{j}\frac{v_{j}}{p_{j}}\nabla^{2}w\left(\left|x_{i}-x_{j}\right|\right). \end{array}$$

The velocity of particle varies between each other and the forces rely on velocity uniqueness not on absolute velocities. There is a natural way to symmetrize the viscosity forces via uniqueness which is expressed as

(13) $$\begin{array}{c} f_{i}^{\text{velocity}}=\mu \underset{j}{\sum }m_{j}\frac{v_{i}+v_{j}}{2p_{j}}\nabla^{2}w\left(\left|x_{i}-x_{j}\right|\right). \end{array}$$

This equation is solved once the neighboring particle of *i* moves from its frame of reference. Particle *i* can be intensified and accelerated in a direction of the virtual speed of its surroundings.

### 3.4. Extreme Expression Generator

The previous sections underscored the creation of sweat and tears via SPH and particle system methods. Now we turn our attention to emphasize the most fundamental issue of generating sweat and tears and their functional dependence on facial mesh. Therefore, the contribution is based on the creation of extreme expression such as fear and happiness which are common in virtual human emotions. Consequently, the facial animation operation with one of the two techniques which is to simulate actual sweat or tears is highlighted.

### 3.5. Facial Skin Color (Blushing)

As mentioned before, the facial skin colors are often used as factors to determine the age or ethnicity. Besides, it serves as a vital element in the appearance, allure, assessment of health status, and attractiveness. Until now, a lot of the data acquired for the skin color and its changes are based on the melanin and hemoglobin distribution. However, few studies focused on the blood oxygenation level that significantly affects the distribution of the hemoglobin which is responsible for the facial color alterations [23, 35–37]. We use the pulse oximeter system and the 3D skin analyzer to measure the effects of oxygenation of the facial skin color appearance.

Following the ideas of Donner and Jensen [38] a modified spectral shading model is developed for rendering human skin color (blushing) based blood oxygenation level. Using [38, 39], the total spectral absorption of the dermis is defined as a combination of absorption from hemoglobin (oxy/deoxy) and small scale structures given by

(14) σaderm(λ)=Ch(γσaoxy(λ)+(1−γ)σadeoxy(λ))+(1−Ch)σabaseline,

where *C*_*h*_ is the fractional amount of hemoglobin in the dermis and *λ* is the wavelength of light in nanometers. The spectral absorption coefficients of oxygenation and deoxygenation are *σ*_*a*_^oxy^ and *σ*_*a*_^deoxy^, respectively (refer to Table 1). The superscript oxy indicates the oxygenation value in the blood and *γ* is the ratio of blood oxygenation between deoxy and oxyhemoglobin. The baseline skin absorption *σ*_*a*_^baseline^ of other small scale tissues in the epidermis and dermis (organelles, cell membranes, fibrils, etc.) is approximated using the expression

(15) $$\begin{array}{c} \sigma_{a}^{\text{baseline}}\left(\lambda \right)=0.0244+8.53e^{-(\lambda -154)/66.2}\;{\text{mm}}^{-1}. \end{array}$$

Two emotional expressions including anger and happiness are simulated using this modified model. Six sets of parameters related to skin appearance are majorly emphasized. The subsurface scattering within the skin and surface reflection from the oily surface are controlled by six physically meaningful parameters including the amount of oxygenation, deoxygenation, hemoglobin, melanin, oiliness, and blend factor between the different types of melanin in the skin. The multipole method for layered materials is used to efficiently calculate the spectral diffusion profiles of two-layered skin. These diffusion profiles are further used during rendering to simulate the subsurface scattering of light within the skin. The reflection of light by the oily skin surface is considered by using the Torrance-Sparrow BRDF model. The model simulation on two skin types (epidermis and dermis) demonstrates the successful reproduction of the spectral reflectance characteristics of human skin color (blushing).

## 4. Implementation

A study was piloted to assess the effect of the sweating model on the sensitivity of fear and tears on the happiness. The sweat and tear expressions are simulated based on particle system and SPH method together with FACS containing AUs.

### 4.1. Facial Action Coding System (FACS) and Action Units

FACS describes facial behaviors based only on facial muscle. The manual version of FACS technique on each change of facial muscle for the appearance was first published in 1978. The facial anatomy analysis exhibits the conduct of human face muscles on the tongue and jaw activities. The FACS begins with facial actions without mentioning the facial behaviors [14]. The facial AUs are aligned to all actions and entail numerous facial muscles. In an expression, the muscles involved are not well distinguished because each of them is distributed into many AUs for explaining autonomous actions of various muscle parts. FACS categorizes the human face into 46 AUs symbolizing different muscle action or a muscle group signifying a single facial position. The lower bound of standard AUs cannot be reduced into unimportant one. Accurate sorting of facial AUs provides the ability to copy every facial muscle activity [15, 40]. The generation of new facial expression is based on the blending of AUs. Originally, the FACS did not ensure the function in achieving the facial animations to describe facial actions. The FACS supports animators to fabricate realistic facial expressions considering every possible facial animation unit prepared from the human face by measuring head and eye positions [17]. The FACS was studied on the muscles movement prior to the examination of the muscle openly. The facial expressions are complicated because a single muscle in isolation cannot induce any expression when the response is cooperative. Therefore, the system being very composite requires a clear comprehensive study for each muscle exclusively [16, 41].

Precisely, the FACS explains most expressions of facial action coding by offering dependable face codes on foreheads, eyelids, and eyebrows. Although FACS overcomes the limits of interpolation, innovative techniques of facial animation are still in demand. This situation allows developing similar techniques based on muscle or simulated muscle-based approaches. Tables 2 and 3 summarize the AUs which reveal realistic facial expression produced via FACS.

**Figure 2:**
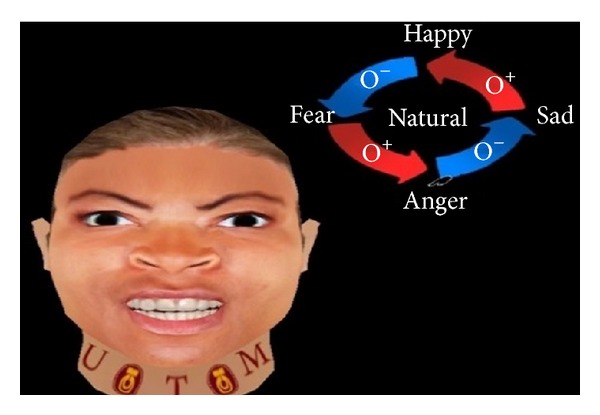
Generated expression for anger with blushing using FACS.

**Figure 3:**
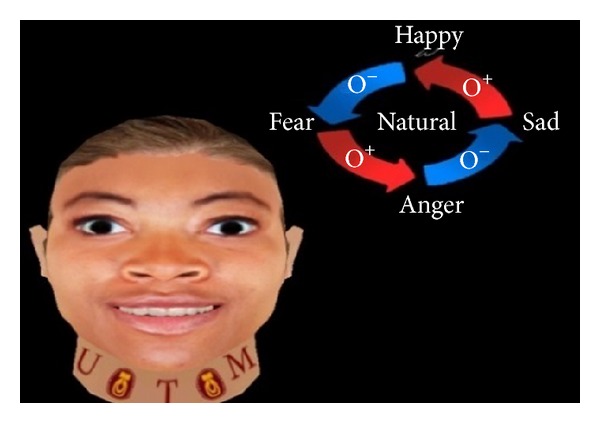
Generated expression for happiness with blushing using FACS.

**Figure 4:**
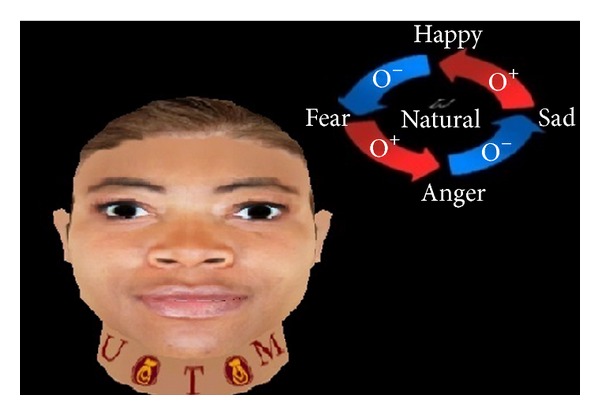
Creation of natural expression using FACS.

**Figure 5:**
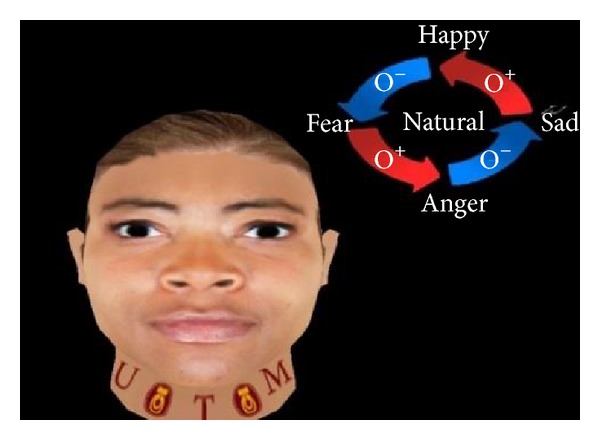
Generation of sad expression using FACS.

**Figure 6:**
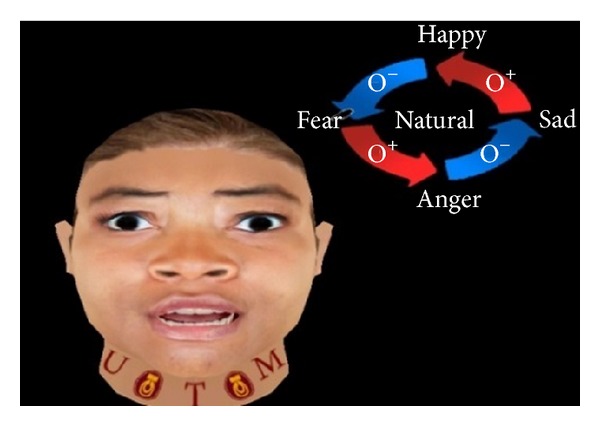
Creation of fear expression using FACS.

The present work applies FACS to create basic expressions such as angry, happy, sad, and fear. Figures 2, 3, 4, 5, and 6 demonstrate the four facial expressions for virtual human including the blushing for anger and happy expressions (without sweating or tears) together. A comparison is further made with the natural one.

### 4.2. Sweating Simulation

**Figure 7:**
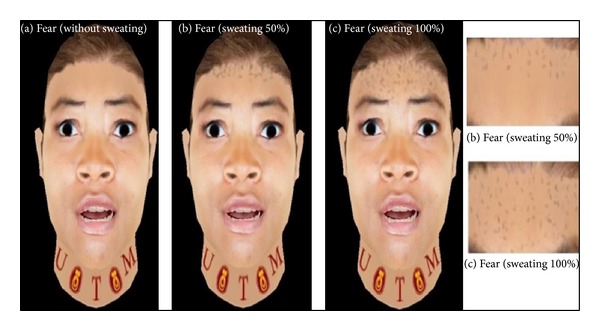
Simulation of sweating due to fear in real-time based on the particle system and SPH method.

Upon blending the facial animation with the fluids generator, integration with the particle system and SPH method is made to generate the sweat and controlled flow on forehead surface. The sweating of fear expression is generated in real-time to represent the extreme expression of virtual avatar.  Figure 7(a) shows the fear expression without sweating. The simulation of sweating is concurrently performed with particle system and SPH method as depicted in Figures 7(b) and 7(c).

### 4.3. Tears Simulation

**Figure 8:**
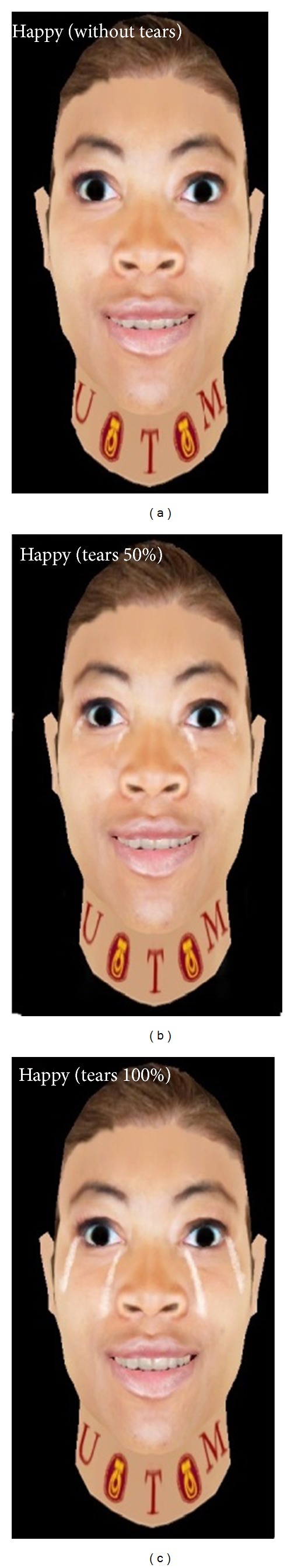
Simulation of tears due to happiness in real-time based on the particle system and SPH method.

As before, the fluid generator reacts with the particle system and SPH method for generating tears in controlling the flows on the eyes surface area corresponding to the strong happy expression for virtual avatar. Tears are created in real-time to represent the extreme expression for virtual avatar.  Figure 8(a) shows the happy expression without tears. The simulation of tears is concurrently performed with particle system and SPH method as depicted in Figures 8(b) and 8(c).

## 5. Conclusions

A novel technique for the creation of realistic human facial expressions is introduced by combining the effects of sweat and tears with the skin color. Generation of extreme appearances such as scared awaiting sweating while happy fit for tears (cry) and blushing (anger and happiness) is the issue. This approach is capable of creating realistic animation expression by producing sweat and tears. Pulse oximeter and the 3D skin analyzer are used to determine the influences of oxygenation on altering skin color appearance. The methods and expressions for creating animated and realistic fluid sweat and tears based on the particle system and SPH method are emphasized. The facial skin appearance is found to be influenced by the physical and physiological state of it. Extreme appearances of sweating with tears and blushing are generated to accomplish the 3D games and the actual facial animation simulation. Detailed animation and realistic facial animation expression by utilizing the same techniques are applied to create the facial expressions. The pragmatic facial expression animation on virtual human by taking into account the changes in facial skin coloration is achieved. Creation of better facial animation model with color, sweat, and tears classification is demonstrated. A correlation between realistic facial color generation and oxygenation and deoxygenation effects of hemoglobin is established. To the best of our knowledge, prior to this model the effects of sweat and tears in conjunction with the color were not integrated into a single animation model to create realistic facial expressions of 3D avatar. The use of FACS on individual faces gives the ability to express and choose any model or animation. The classification of facial actions into minor unit termed as action units has enabled the facial expressions to be displayed in a desirable manner. This continually falls and changes the shape as time upsurges. The proposed framework is capable of simulating actual tears, sweat, and blushing via the integration of facial animation system with the particle system and SPH method. Our unique combination influencing the facial animation framework may allow the animators to control the sweating and tears motions via a set of parameters subjected to the FACS standard.

## Figures and Tables

**Table 1:** Physiological parameters of oxygenation in the blood obtained by us.

Parameter	Description	Typical range
*C*_*h*_	Hemoglobin fraction	0–0.1
Oxy	Oxygenation	0.9–0.99
Deoxy	Deoxygenation	0.01–0.1

**Table 2:** Action units used to represent four basic expressions [17].

AUs	FACS name	AUs	FACS name	AUs	FACS name
6	Check raiser	23	Lip tightener	15	Lip corner depressor
1	Inner brow raiser	26	Jaw drop	5	Upper lid raiser
17	Raiser chin	20	Lip stretcher	9	Nose wrinkle
4	Brow lower	14	Dimpler	16	Lower lip depressor
10	Upper lip raiser	2	Outer brow raiser	12	Lid corner puller

**Table 3:** Example of AU sets for four expressions [17].

Expressions	Involved AUs
Anger	AU (26 + 4 + 17 + 10 + 9 + 20 + 2)
Fear	AU (2 + 4 + 5 + 26 + 15 + 20 + 1)
Happiness	AU (14 + 12 + 6 + 1)
Sadness	AU ( 23 + 1 + 15 + 4)
